# Non-linear ion transport in nanopores for the design of ultracapacitive ionic memristors

**DOI:** 10.1093/nsr/nwae437

**Published:** 2024-12-14

**Authors:** Panlong Li, Stefan Kaskel

**Affiliations:** Inorganic Chemistry Center I, Technische Universität Dresden, Germany; Inorganic Chemistry Center I, Technische Universität Dresden, Germany; Fraunhofer IWS, Germany

In today's data-driven world, energy consumption and CO_2_ emissions resulting from digitalization and computation are steadily increasing, which are significant challenges for our society. In the course of natural evolution, biological nervous systems efficiently operate via numerous ions and neurotransmitters to perform parallel information processing and in-memory computing tasks with ultra-low energy consumption. Hence, mimicking ion-based information processing strategies inspired by natural evolution, rather than relying on electrons and holes, presents a vital direction to surpass the limitations of the state-of-the-art von Neumann architectures [1].

Recently, an ultracapacitive strategy was developed for ion manipulation, enabling the construction of various ultracapacitive ion-based devices (i.e. ultracapacitive iontronics). Examples include ultracapacitive ionic diodes [2] and ionic transistors [3]. Moreover, ionic diodes performed simple logic functions such as OR and AND gates, demonstrating their functionality in fundamental logic circuits [4]. However, these applications are still based on binary logic (‘on’ and ‘off’) and cannot compete with existing electronics. Only by adopting a biological neural strategy and designing ion-based devices with neuromorphic functionality can the limitations of von Neumann architectures be overcome. Due to their neuromorphic features, ionic memristors have the potential to mimic the computing characteristics of biologic neural systems [5].

**Figure 1.**
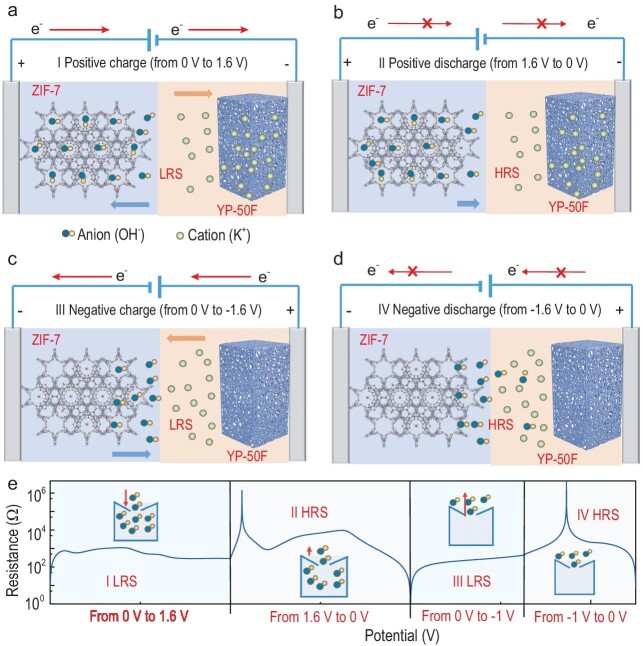
The working mechanism of the CAPistor. (a, b) Increased forward bias (positive charge from 0 to 1.6 V) and decreasing forward bias (positive discharge from 1.6 to 0 V). (c, d) Increased reverse bias (negative charge from 0 to −1.6 V) and decreasing reverse bias (negative charge from −1.6 to 0 V) with ZIF-7 as the working electrode and YP-50F as the counter electrode. (e) The corresponding resistive switching behaviors of the CAPistor. Reproduced from ref. [6] with open access.

Yan's team introduced the supercapacitor-memristor (termed ‘CAPistor’) as an ultracapacitive ionic memristor with a high ratio of high-resistance state (HRS) and low-resistance

state (LRS) (Fig. 1) [6]. The memristive characteristic relies on a hysteresis effect in the ionic conductivity of hydroxide anions, caused by the enrichment and dissipation of the hydroxide anions within the nano-channels of zeolitic imidazolate framework ZIF-7 electrodes at varying voltages (so-called non-linear ion transport). The working mechanism of the CAPistor can be explained as follows.

Increased forward bias (positive charging from 0 to 1.6 V in Fig. 1a): when a gradually increasing forward bias is applied to the ZIF-7 electrode, hydroxide ions are electrosorbed in the ZIF-7 pores, and potassium ions enter the YP-50F carbon pores, leading to a pronounced current response (i.e. LRS).

Decreasing forward bias (discharge in positive window from 1.6 to 0 V in Fig. 1b): as the forward bias decreases, only a small number of hydroxyl ions are desorbed from ZIF-7 pores. Therefore, an ionic concentration equilibrium within the pores and in the bulk solution is built along with the lag of the external electric field, leading to a small current response (i.e. HRS).

Increased reverse bias (negative charging from 0 to −1.6 V in Fig. 1c): when the ZIF-7 electrode becomes negatively polarized, the hydroxide ions in ZIF-7 pores overcome the energy barrier, generating a noticeable current response (i.e. LRS).

Decreasing reverse bias (negative charging from −1.6 to 0 V in Fig. 1d): as the reverse bias decreases, no ion movement in ZIF-7 pores leads to an open circuit with no current response (i.e. HRS).

As a consequence, the ion flux-induced changes in the supercapacitor's conductivity enable the realization of memristive characteristics (Fig. 1e).

The remarkable memristive characteristic in supercapacitors obtained by Yan's team extends the family of ultracapacitive iontronic devices with the addition of an ultracapacitive ionic memristor, which has the potential to be utilized in the realm of biomimetic nanofluidic devices and neuromorphic computing. This field is truly in its infancy. Novel experimental and simulation methods are yet to be developed for characterizing and monitoring, *in situ*, the states of ions confined in nanopores. A wide field has been opened, expanding the application scenarios of ultracapacitive devices into new areas such as bioniterfacing, neuromorphic computing and artificial intelligence.
